# Cell adhesion and intracellular calcium signaling in neurons

**DOI:** 10.1186/1478-811X-11-94

**Published:** 2013-12-13

**Authors:** Lifu Sheng, Iryna Leshchyns’ka, Vladimir Sytnyk

**Affiliations:** 1 School of Biotechnology and Biomolecular Sciences, The University of New South Wales, Sydney, New South Wales 2052, Australia

**Keywords:** Cell adhesion molecule, Neurons, Calcium, Voltage dependent Ca^2+^ channel, Neurite outgrowth

## Abstract

Cell adhesion molecules (CAMs) play indispensable roles in the developing and mature brain by regulating neuronal migration and differentiation, neurite outgrowth, axonal fasciculation, synapse formation and synaptic plasticity. CAM-mediated changes in neuronal behavior depend on a number of intracellular signaling cascades including changes in various second messengers, among which CAM-dependent changes in intracellular Ca^2+^ levels play a prominent role. Ca^2+^ is an essential secondary intracellular signaling molecule that regulates fundamental cellular functions in various cell types, including neurons. We present a systematic review of the studies reporting changes in intracellular Ca^2+^ levels in response to activation of the immunoglobulin superfamily CAMs, cadherins and integrins in neurons. We also analyze current experimental evidence on the Ca^2+^ sources and channels involved in intracellular Ca^2+^ increases mediated by CAMs of these families, and systematically review the role of the voltage-dependent Ca^2+^ channels (VDCCs) in neurite outgrowth induced by activation of these CAMs. Molecular mechanisms linking CAMs to VDCCs and intracellular Ca^2+^ stores in neurons are discussed.

## Review

Cell adhesion molecules (CAMs) expressed on the neuronal cell surface play crucial roles in neuronal migration, axonal fasciculation, and neurite outgrowth during brain development. They also play an important role in regulation of synaptic plasticity in adult brain and axonal regeneration in injured nervous system [1-6].

Functions of CAMs are induced in response to their binding to ligands presented either in the soluble form or on membranes of other cells or on artificial surfaces, a process which is often called CAM activation. CAM activation induces a number of intracellular signaling cascades, which are essential for CAM-mediated functions (for extensive review see [5,7-11]). Among signaling cascades activated by CAMs, changes in intracellular Ca^2+^ levels have been documented to occur in neurons in response to activation of virtually all families of CAMs, including the three main families comprising the immunoglobulin superfamily (IgSF), cadherins and integrins.

Intracellular Ca^2+^ serves as a secondary signaling messenger with Ca^2+^ channels at the neuronal cell surface and internal Ca^2+^ stores regulating intracellular Ca^2+^ concentrations in neurons and other cells. Intracellular Ca^2+^ has critical roles in all aspects of neuronal development including neurite elongation and neuronal growth cone motility in developing neurons [12-17].

Intracellular signaling cascades activated by CAMs in response to ligand binding induce a number of physiologically important responses in neurons, among which changes in neurite outgrowth are probably the best characterized for CAMs of different families [5,7,11,18]. In this review, we systematically analyze studies reporting changes in intracellular Ca^2+^ levels in response to activation of IgSF CAMs, cadherins and integrins in neurons. We also analyze the experimental evidence supporting the involvement of the cell surface Ca^2+^ channels and intracellular Ca^2+^ stores in intracellular Ca^2+^ changes induced by CAMs of these families, and review the data showing the effects of Ca^2+^ channel inhibitors on CAM-induced neurite outgrowth.

### Changes in intracellular Ca^2+^ levels induced by activation of CAMs of the immunoglobulin superfamily (IgSF)

CAM-induced increases in intracellular Ca^2+^ levels were first documented in response to activation of the neural cell adhesion molecule (NCAM) and L1, both members of the IgSF (Table 1). NCAM mediates homophilic adhesion, i.e. extracellular domains of NCAM molecules on cell surface membranes of adjacent cells bind to each other. It functions in the developing nervous system by regulating neuronal migration and differentiation and also plays an important role in adult brain by regulating memory formation and brain plasticity [19,20]. Application of purified NCAM, which binds to NCAM at the cell surface, induced an increase in intracellular Ca^2+^ levels in small cerebellar neurons [21]. Similar results were obtained with artificial ligands of NCAM, such as peptide ligands of NCAM, which bind to the extracellular domain of NCAM and which have been shown to induce an increase in intracellular Ca^2+^ levels in PC12 cells and rat hippocampal neurons loaded with a Ca^2+^ indicator Fura-2 acetoxymethyl ester (AM) [22,23]. The effects of antibodies against the extracellular domain of NCAM used as an artificial NCAM ligand were analyzed in several studies and have been shown to increase intracellular Ca^2+^ levels in PC12 cells and in small cerebellar neurons as measured using fluorimetry and a Ca^2+^ indicator Quin-2AM [24,25]. In another study, polyclonal but not monoclonal antibodies against the extracellular domain of NCAM have been shown to induce an increase in intracellular Ca^2+^ levels in dorsal root ganglion neurons but not in small cerebellar neurons loaded with Fura-2AM [21], suggesting that the type of the antibodies used can influence the effects of the antibody on intracellular Ca^2+^ levels.

**Table 1:** **An overview of CAMs, activation of which induces an increase in intracellular Ca**^**2+ **^**levels**

**CAMs**	**Method/Ca**^**2+ **^**indicator**	**Ligand, concentration**	**Cell types (localization)**	**Effect on Ca**^**2+**^	**Inhibitors tested (effect on ligand induced Ca**^**2+ **^**increase)**	**References**
**IgSF**						
NCAM	Fluorimetry/Quin-2AM	Polyclonal NCAM antibodies, 0.4-1 mg/ml	PC12 cells	**↑**	Verapamil (full inhibition)	[24]
					Diltiazem (full inhibition)	
					Nifedipine (no effect)	
	Microscopy/Fura-2AM	Synthetic peptide ligand of NCAM ectodomain, 50 μM	PC12-E2 cells	**↑**	Not tested	[22]
	Fluorimetry/Quin-2AM	Fab fragments of monoclonal NCAM antibodies (H28), 0.1-0.2 mg/ml	PC12 cells	No effect	Not tested	[24]
	Microscopy/Fura-2AM	Polyclonal NCAM antibodies, 0.3-0.5 mg/ml	PC12 cells	**↑**	Not tested	[21]
	Microscopy/Fura-2AM	Monoclonal NCAM antibodies, 0.1 mg/ml	PC12 cells	No effect	Not tested	[21]
	Microscopy/Fura-2AM	NCAM antibodies, 0.5 mg/ml	Chick ciliary ganglion neurons	No effect	Not tested	[39]
	Microscopy/Fura-2AM	Polyclonal NCAM antibodies, 0.3-1 mg/ml	Mouse dorsal root ganglion neurons	**↑**	Not tested	[21]
	Microscopy/Fura-2AM	Monoclonal NCAM antibodies, 0.1 mg/ml	Mouse dorsal root ganglion neurons	No effect	Not tested	[21]
	Microscopy/Fura-2AM	Polyclonal NCAM antibodies, 0.5 mg/ml	Mouse small cerebellar neurons	No effect	Not tested	[21]
	Microscopy/Fura-2AM	Monoclonal NCAM antibodies, 0.1 mg/ml	Mouse small cerebellar neurons	No effect	Not tested	[21]
	Microscopy/Fura-2AM	Purified NCAM from mouse brain, 10 μg/ml	Mouse small cerebellar neurons	**↑**	Not tested	[21,25]
	Microscopy/Fura-2AM	Recombinant fragments of NCAM ectodomain, 0.8 μM	Mouse small cerebellar neurons	**↑**	Not tested	[25]
	Microscopy/Fura-2AM	Monoclonal NCAM antibodies, 30 μg/ml	Mouse cortical neurons (soma)	No effect	Not tested	[29,30]
	Microscopy/Fura-2AM	Synthetic peptide ligand of ectodomain NCAM, 54 μM	Rat hippocampal neurons	**↑**	Not tested	[22]
		Polyclonal NCAM antibodies, 1 mg/ml	Rat hippocampal neurons	**↑**	Not tested	[22]
	Microscopy/ Fura-2AM or Fluo-4AM	Synthetic peptide ligand of NCAM, 12–35 μM	Rat hippocampal neurons (soma)	**↑**	Nifedipine (partial inhibition)	[23]
					Mibefradil (partial inhibition)	
					Pimozide (full inhibition)	
					ω-conotoxin (no inhibition)	
					Agatoxin (no inhibition)	
					Loe908 (partial inhibition)	
					SKF-96365 (partial inhibition)	
L1	Fluorimetry/Quin-2AM	Polyclonal L1 antibodies, 0.4-1 mg/ml	PC12 cells	**↑**	Not tested	[21,24]
	Microscopy/Fura-2AM	Polyclonal L1 antibodies, 0.3-1 mg/ml	Mouse dorsal root ganglion neurons	**↑**	Verapamil (no effect)	[21]
					Diltiazem (no effect)	
					Cd^2+^/Ni^2+ ^(no effect)	
	Microscopy/Fura-2AM	Recombinant ectodomain of L1 (L1-Fc), 10 μg/ml	Rat dorsal root ganglion neurons (growth cones)	**↑**^a^	Nifedipine (full inhibition)^a^	[37]
					Conotoxin (partial inhibition)^a^	
	Microscopy/Fura-2 dextran	L1 expressed by 3 T3 cells	Rat dorsal root ganglion neurons (growth cones)	No effect	Not tested	[31]
	Whole cell patch-clamp	Monoclonal L1 antibodies recognizing glycosylated L1, 7.5-30 μg/ml	Mouse dorsal root ganglion neurons	**↑**	Nifedipine (full inhibition)	[30]
					Cd^2+^ (full inhibition)	
	Microscopy/Fura-2 AM	Polyclonal L1 antibodies, 0.3-0.5 mg/ml	Mouse small cerebellar neurons	**↑**	Verapamil (no effect)	[21]
					Diltiazem (no effect)	
					Cd^2+^/Ni^2+ ^(No effect)	
	Fluorimetry/Quin-2AM; Microscopy/Fura2AM	Purified L1 from mouse brain, 10 μg/ml or 0.8 μM	Mouse small cerebellar neurons	**↑**	Not tested	[21,25,28]
	Fluorimetry/Quin-2AM	Monoclonal L1 antibodies recognizing FNIII type repeats, 100 μg/ml	Mouse small cerebellar neurons	**↑**	Not tested	[28]
	Fluorimetry/Quin-2AM	Monoclonal L1 antibodies recognizing Ig-like domains I-VI, 100 μg/ml	Mouse small cerebellar neurons	No effect	Not tested	[21,28]
	Microscopy/Fura-2AM,	Monoclonal L1 antibodies recognizing glycosylated L1, 7.5-30 μg/ml	Mouse cortical neurons (soma)	**↑**	Nifedipine (full inhibition)	[29,30]
	Whole cell patch-clamp					
					Cd^2+^ (full inhibition)	
Np55	Microscopy/Fluo-4AM	Soluble recombinant ectodomain of Np55, 15 μM	Rat hippocampal neurons	**↑**	Not tested	[35]
			(synaptic areas)			
Np65	Micriscopy/Fluo-4AM	Soluble recombinant ectodomain of Np65, 15 μM	Rat hippocampal neurons	**↑**	Not tested	[34]
			(synaptic areas)			
	Micriscopy/Fluo-4AM	Syntetic peptide ligand of Np65 enplastin, 7–15 μM	Rat hippocampal neurons	**↑**	Not tested	[34]
			(synaptic areas)			
NgCAM	Microscopy/Fluo-3AM; Fura-2AM	Purified chicken NgCAM, 1.2 μg/ml	Neostriatal subependymal zone neurons of adult zebra finch	**↑**	Nifedipine (full inhibition)	[32]
					ω-conotoxin (partial inhibition)	
	Microscopy/Fluo-3AM; Fura-2AM	Polyclonal NgCAM antibodies, 100 μg/ml	Neostriatal subependymal zone neurons of adult zebra finch	**↑**	Nifedipine (full inhibition)	[32]
					ω-conotoxin (partial inhibition)	
LAMP	Microscopy/Fluo-3AM	Soluble recombinant LAMP, 30 μg/ml	Rat hippocampal neuron	**↑**	Nifedipine (full inhibition)	[36]
					ω-conotoxin (no inhibition)	
	Microscopy/Fluo-3AM	Soluble recombinant LAMP, 30 μg/ml	Visual cortex neurons	**↑**	Not tested	[36]
Thy-1	Microscopy /Fura-2AM	Fab fragments of monoclonal Thy-1 antibodies, 10 μg/ml	PC12 cells (cytosol)	No effect	Not tested	[68]
**Cadherins**						
N-cadherin	Microscopy/Fura-2AM	Soluble fragments of N-cadherin purified from brain or retina, 10 μg/ml	Chick ciliary ganglion neurons	**↑**	Mixture of diltiazem and ω-conotoxin (no inhibition)	[39]
			(soma, growth cones)			
	Whole cell voltage clamp	Recombinant ectodomain of N-cadherin (N-cadherin-Fc), 20 μg/ml	Chick ciliary ganglion neurons	**↑**	Not tested	[41]
	Microscopy/Fura-2AM	Soluble recombinant ectodomain of N-cadherin (N-cadherin-Fc), 50 μg/ml	Chick retinal ganglion cells	No effect	Not tested	[40]
	Microscopy/FFP-18-AM	Soluble recombinant ectodomain of N-cadherin (N-cadherin-Fc), 50 μg/ml	Chick retinal ganglion cells (subplasma membrane of growth cones)	**↑**	Mixture of nifedipine and ω-conotoxin (partial inhibition)	[40]
Celsr2/Celsr3	Microscopy/Fura-2AM	Soluble recombinant cadherin repeats of Celsr2/Celsr3, 1 μg/ml	Rat hippocampal neurons	**↑**	Not tested	[38]
**Integrins**						
β Integrin	Microscopy/Fura-2AM, whole cell voltage clamp	RGD peptide (cGRGDSPA), 1 μM	*L. stagnalis* CNS motoneurons	**↑**	Not tested	[48]
			(soma)			
	High-speed microscopy/Fluo-4AM	Synthetic RGD peptide (RGDS), 0.5-1 μM	*Xenopus* spinal neurons (growth cones)	**↑**	Not tested	[44]
	Microscopy/Fura-2AM	Soluble Laminin, 20 μg/ml	Chick ciliary ganglion neurons (soma)	**↑**	Mixture of diltiazem and ω-conotoxin (no inhibition)	[39]
	Microscopy/Fura-2AM	Laminin, immobilized to the beads, 50 μg/ml	Chick dorsal root ganglion neurons (growth cones)	No effect	Not tested	[43]
	Microscopy/Fura-2AM	Soluble laminin, 20 μg/ml	Surgically isolated filopodia from growth cones of chick dorsal root ganglion neurons	**↑**	Not tested	[43]
	Microscopy/Fura-2AM	RGD peptide (GRGDSP), 10 μM	Mouse cortical neurons (soma and neurites)	**↑**	Gd^3+^ (partial inhibition)	[45]
					Nifedipine (partial inhibition)	
	Whole cell voltage clamp	Polyclonal α5β1 integrin antibodies, 10 μg/ml	Rat basal forebrain neurons	**↑**	Not tested	[47]
	Microscopy/Fura-2AM	Syntetic RGD peptide (GRGDSP), 2.5 mM	Rat cortical neurons	**↑**	Not tested	[46]

^a^effect observed in the presence of Ba^2+^ in the test solution.

CAMs, methods used to detect changes in intracellular Ca^2+^ levels, Ca^2+^ sensitive indicator used in optical recordings, ligands used to activate CAMs, cell type analyzed and subcellular localization of Ca^2+^ changes (if described in the original publication), the effect of CAMs on intracellular Ca^2+^ levels (↑ - indicates an increase), the effect of inhibitors of Ca^2+^ channels on CAM-induced intracellular Ca^2+^ increases, and respective references are listed in the table.

CAMs of the L1 family, including L1, close homolog of L1 (CHL1), neurofascin and Neuron-glia cell adhesion molecule (NgCAM), also mediate homophilic adhesion and play a prominent role in the developing central nervous system (CNS) [26,27]. Fluorimetric observations of Quin-2AM loaded cells and microscopical recording of Fura-2AM loaded neurons showed that purified or recombinant L1 and antibodies against the extracellular domain of L1 induce an increase in intracellular Ca^2+^ levels in PC12 cells [24], small cerebellar neurons [21,25,28], cortical neurons [29,30], and dorsal root ganglion neurons [21]. Ca^2+^ influx in response to L1 activation has also been observed using voltage patch clamp recordings in cortical neurons [30]. In another study, however, activation of L1 had no effect on intracellular Ca^2+^ levels in Fura-2AM loaded growth cones of dorsal root ganglion neurons [31]. Interestingly, the effect of L1 antibodies depended on the epitope recognized, with one study reporting an increase in intracellular Ca^2+^ levels in neurons incubated with a monoclonal antibody against an epitope within fibronectin type III repeats of L1, but not in neurons incubated with the monoclonal antibodies against epitopes within immunoglobulin domains [28], suggesting that differences in L1 ligands used may contribute to differences in the effects on intracellular Ca^2+^ levels. Similar to L1, exposure to immunopurified NgCAM or anti-NgCAM antibodies induced an increase in intracellular Ca^2+^ levels in neurons from the brains of songbird zebra finch loaded with a Ca^2+^ indicator Fluo-3AM and analyzed by confocal microscopy [32]. While the role of Ca^2+^ in signaling induced by another member of this family, CHL1, has been recently suggested [33], the direct evidence that CHL1 can also induce an increase in intracellular Ca^2+^ levels is still missing.

In addition to NCAM and L1 family members, activation of the immunoglobulin superfamily cell adhesion molecules neuroplastin (Np) and limbic system-associated membrane protein (LAMP) has also been shown to induce increases in intracellular Ca^2+^ levels. Np mediates cell-to-cell adhesion via homophilic interactions and is expressed as two isoforms named according to the molecular weight Np55 and Np65. Application of recombinant ectodomains of Np55 and Np65 or a mimicking peptide of Np65 induced an increase in intracellular Ca^2+^ levels in synapses of cultured hippocampal neurons loaded with a Ca^2+^ indicator Fluo-4AM [34,35]. Soluble recombinant LAMP has been shown to induce increases in intracellular Ca^2+^ in hippocampal neurons and neurons from visual cortex loaded with Fluo-3AM [36].

Experiments with inhibitors of various types of Ca^2+^ channels indicate that changes in intracellular Ca^2+^ levels in response to activation of IgSF CAMs can be mediated by several classes of VDCCs. Inhibitors of L- and T-type VDCCs reduced the increase in intracellular Ca^2+^ levels observed in response to NCAM activation in cultured hippocampal neurons [23] and PC12 cells [24]. Pimozide, an inhibitor of T-type VDCCs, was more potent in inhibiting the NCAM-induced Ca^2+^ response when compared to nifedipine, an inhibitor of L-type VDCCs, in cultured hippocampal neurons [23]. Fluorometric Ca^2+^ measurements showed that inhibitors of nonselective cation channels also reduced NCAM-dependent Ca^2+^ influx in Fura-2AM loaded neurons, suggesting that these channels are also activated in response to NCAM ligands [23]. Nifedipine fully blocked and ω-conotoxin, an inhibitor of N-type VDCCs, partially blocked L1-dependent increase in intracellular Ca^2+^[29,30,37], while in another study inhibitors of L-type VDCCs verapamil and diltiazem failed to block the L1-dependent Ca^2+^ influx in mouse dorsal root ganglion neurons and small cerebellar neurons [21]. Nifedipine and ω-conotoxin blocked NgCAM-induced Ca^2+^ influx [32]. The LAMP-induced intracellular Ca^2+^ increases in hippocampal neurons were fully inhibited by nifedipine but not ω-conotoxin [36]. Altogether, these data indicate that different members of IgSF act at different VDCCs in a tissue specific manner.

Not only inhibitors of cell surface Ca^2+^ channels, but also depletion of the internal Ca^2+^ stores by incubation with thapsigargin, a specific inhibitor of the sarcoendoplasmic reticulum Ca^2+^-ATPase, inhibited an increase in intracellular Ca^2+^ levels in response to activation of NCAM in cultured hippocampal neurons [23,38] and in response to NgCAM activation in cultured forebrain neurons [32], indicating that Ca^2+^ influx at the cell surface membrane is accompanied by the release of Ca^2+^ from the internal stores. Internal Ca^2+^ stores have also been suggested to contribute to intracellular Ca^2+^ increases in response to activation of Np55 [35].

### Changes in intracellular Ca^2+^ levels induced by activation of cadherins

Optical recordings of chick ciliary ganglion neurons loaded with Fura-2AM showed that application of soluble fragments of N-cadherin purified from brain resulted in an increase in intracellular Ca^2+^ levels in growth cones and cell bodies of neurons [39]. Increased steady state levels of intracellular Ca^2+^ were also recorded in growth cones of chick retinal ganglion cells grown on N-cadherin and loaded with a membrane targeted Ca^2+^ indicator, FFP-18AM [40]. Interestingly, when the Fura-2AM reporter was used, steady state levels of intracellular Ca^2+^ were found to be not affected by substrate coated recombinant N-cadherin suggesting that N-cadherin influences predominantly submembrane Ca^2+^ levels [40]. Whole cell voltage clamp recordings also showed that homophilic binding of N-cadherin on neuronal membranes to soluble N-cadherin or N-cadherin overexpressed in Chinese hamster ovary (CHO) cells increases amplitudes of Ca^2+^ currents in ciliary ganglion neurons indicating that homophilic interactions of N-cadherin are sufficient to activate a cellular mechanism that regulates Ca^2+^ influx [41].

Similarly to members of IgSF, experiments with inhibitors of various types of Ca^2+^ channels suggest that VDCCs play an important role in cadherin-induced increases in intracellular Ca^2+^ levels. In particular, co-application of nifedipine and ω-conotoxin partially reduced the increase in intracellular Ca^2+^ levels in response to N-cadherin activation in retinal ganglion cells [40], suggesting that L- and T-type VDCCs are involved in N-cadherin induced Ca^2+^ influx in neurons. It should be noted, however, that inhibitors of N- and L-type VDCCs ω-conotoxin and diltiazem had no significant effect to N-cadherin-induced Ca^2+^ response in ciliary ganglion neurons [39].

In addition to N-cadherin, increases in intracellular Ca^2+^ levels have been shown to occur in response to activation of atypical cadherins Celsr2 and Celsr3, which are highly expressed in hippocampal and cortical neurons [42]. Optical imaging of cultured hippocampal neurons loaded with Fura-2AM showed that intracellular Ca^2+^ levels increased in response to recombinant cadherin repeats of Celsr3 and Celsr2 with a more pronounced effect observed in response to activation of Celsr2 when compared to Celsr3 [38]. This increase in intracellular Ca^2+^ levels in response to activation of Celsr2 or Celsr3 was inhibited by thapsigargin, indicating that intracellular Ca^2+^ stores play also a role in Ca^2+^ increases mediated by cadherin family members [38].

### Changes in intracellular Ca^2+^ levels induced by activation of integrins

Modest increases in intracellular Ca^2+^ levels were reported to occur in response to exposure of neurons to natural ligands of integrins. Optical recordings showed that laminin induced an increase in intracellular Ca^2+^ levels in growing Fura-2AM loaded chick ciliary ganglion neurons [39], and in the surgically isolated filopodia of growth cones of chick dorsal root ganglion neurons [43]. Much larger increases in intracellular Ca^2+^ levels were observed in response to integrin ligand Arginine-Glycine-Aspartic acid (RGD)-sequence containing peptides. Optical recordings of Fluo-4AM loaded cultured *Xenopus* spinal neurons showed that incubation with soluble RGD peptides elevated intracellular Ca^2+^ levels in growth cones and increased filopodial Ca^2+^ transient frequency [44]. Similar results were obtained with adult cortical neurons, in which fibronectin application has produced moderate increases in intracellular Ca^2+^ levels while larger responses were observed in neurons treated with RGD-containing peptides [45,46]. Increased Ca^2+^ currents induced by activation of integrins using multivalent antibodies against integrins were also observed using whole cell patch clamp recordings in neurons acutely dissociated from the medial septum/diagonal band nucleus of the rat [47]. Both, optical recordings of Fura-2AM loaded cell bodies and whole cell voltage clamp recordings showed that RGD peptides increased depolarization induced increases in intracellular Ca^2+^ levels in motoneurons isolated from the CNS of the pond snail *L. stagnalis*[48]. It should be noted, however, that high concentrations of RGD peptides used in some of the previous studies [46] have also been shown to induce integrin-independent increases in intracellular Ca^2+^ levels, such as via activation the N-methyl-D-aspartate (NMDA) receptors in an integrin-independent manner [49]. Therefore, contribution of integrin-independent sources of Ca^2+^ to overall increases in intracellular Ca^2+^ levels in studies using RGD peptides cannot be fully excluded.

Integrin β-dependent increases in intracellular Ca^2+^ levels were partially blocked by nifedipine and gadolinium III (Gd^3+^), a broad spectrum VDCC inhibitor, in cortical neurons [45]. However, a mixture of diltiazem and ω-conotoxin did not affect the laminin-induced Ca^2+^ increases in somata of chick ciliary ganglion neurons [39]. Depletion of intracellular Ca^2+^ stores and inhibitors of the ryanodine receptor (RyR) and inositol 1,4,5-triphosphate gated receptor (IP3R), channels through which Ca^2+^ in intracellular stores is released into the cytosol, also reduced but did not eliminate increases in intracellular Ca^2+^ levels in response to RGD-containing integrin ligand peptides in cortical neurons [45]. Therefore, Ca^2+^ influx via VDCCs and Ca^2+^ release from internal stores can both contribute to the elevation of intracellular Ca^2+^ levels in response to integrin activation.

### Changes in intracellular Ca^2+^ levels induced by activation of other CAMs

Changes in intracellular Ca^2+^ levels have also been reported for other neuronal cell surface molecules involved in neuronal adhesion, notably for amyloid precursor protein (APP) and cellular prion protein (PrP). Optical recordings of B103 rat neuroblastoma cells transfected with APP and loaded with Fluo-4AM showed an increase in intracellular Ca^2+^ levels in response to incubation with amyloid beta (Aβ), an APP-derived toxic peptide accumulating in brains of Alzheimer’s disease patients. Since no changes in intracellular Ca^2+^ levels in response to Aβ occured in cells non-transfected with APP, it was proposed that binding of Aβ to APP induced Ca^2+^ influx in these cells [50]. Dysregulation of Ca^2+^ signaling has been also found in astrocytes from mice missing APP [51].

An increase in intracellular Ca^2+^ levels have been observed in synaptosomes incubated with recombinant PrP, while function blocking antibodies against PrP inhibited depolarization induced Ca^2+^ influx via synaptosomal VDCCs, indicating that PrP also plays a role in regulation of intracellular Ca^2+^ levels [52]. PrP dependent Ca^2+^-influx has been shown to occur in response to such ligands of PrP as laminin and stress-inducible protein 1 in dorsal root ganglion neurons loaded with Fluo-3AM [53]. Reduced depolarization induced Ca^2+^ influx has been observed using Fura-2AM and a Ca^2+^ indicator Calcium Green-5N in cerebellar granule cells and hippocampal CA1 neurons from PrP deficient mice, respectively [54,55]. Both submembrane and intracelluar levels of Ca^2+^ were affected by PrP deficiency [55].

Reduced Ca^2+^ currents have been also recorded in mice deficient in α-neurexin [56], indicating that neurexin-neuroligin adhesion complexes are also involved in regulation of intracellular Ca^2+^ levels in neurons. Whether binding of α-neurexins to neuroligins stimulates Ca^2+^ influx into neurons remains to be investigated.

### The effect of VDCC inhibitors on neurite outgrowth induced by activation of IgSF CAMs, cadherins and integrins

VDCCs have been shown to play a multitude of roles in the developing and adult brain being involved in a number of signaling pathways. The role of different types of VDCCs in various brain functions is beyond the scope of this review and we refer the reader to several recent excellent reviews on this subject [57-63]. Below, we summarise current evidence implicating VDCCs in CAM-induced neurite outgrowth.

Analysis of studies investigating effects of various inhibitors of Ca^2+^ channels on CAM-induced neurite outgrowth is summarized in Table 2. A study by Doherty and colleagues [64], which demonstrated that inhibitors of L-type and N-type VDCCs inhibit NCAM-mediated neurite outgrowth from PC12 cells in an additive manner, was the first to show the fundamental role of VDCCs in neurite outgrowth mediated by CAMs. NCAM-dependent neurite outgrowth has been also shown to be partially inhibited by the inhibitors of N-, L-, and P/Q-type VDCCs in cultured hippocampal neurons [22,65]. Inhibitors of L-type VDCCs also blocked exocytosis in growth cones induced in response to NCAM activation and required for NCAM-dependent neurite outgrowth [66]. Interestingly and surprisingly, another study showed that inhibitors of T-type VDCCs or inhibitors of nonselective cation channels also completely blocked NCAM-dependent neurite outgrowth in cultured hippocampal neurons [23], suggesting that Ca^2+^ influx via different Ca^2+^ channels is necessary to raise the overall levels of intracellular Ca^2+^ above the threshold required for NCAM-dependent neurite outgrowth. Similarly, L1-dependent neurite outgrowth was blocked by inhibitors of L-type (diltiazem, verapamil, or nifedipine) and N-type (ω-conotoxin) VDCCs in rat cerebellar neurons and PC12 cells [31,67], and partially inhibited by nifedipine, verapamil and diltiazem in mouse small cerebellar neurons [28]. Neurite outgrowth induced by another member of L1 family, CHL1, was fully blocked by application of either an inhibitor of L- or T-type VDCCs [33]. Inhibitors of L- and N-type VDCCs have been also shown to block neurite outgrowth induced by activation of IgSF cell adhesion molecule Thy-1 [31,67,68]. In contrast, neurite outgrowth induced in cultured hippocampal neurons grown on CHO cells overexpressing LAMP was inhibited by blockers of L- but not N-type VDCCs [36]. Altogether, these observations suggest that Ca^2+^ influx via distinct Ca^2+^ channels at the cell surface is required to induce a complete set of molecular changes and responses required for IgSF CAM-dependent neurite outgrowth. This scenario is consistent with the observations showing that VDCCs can activate several independent signaling pathways in growth cones of growing neurites [69].

**Table 2:** **An ovsssserview of the effects of the inhibitors of Ca**^**2+ **^**channels on CAM-mediated neurite outgrowth**

**CAMs**	**Cell types**	**Inhibitors (type of Ca**^**2+ **^**channels)**	**Impact on CAM-mediated neurite outgrowth**	**References**
**IgSF**				
NCAM	PC12 cells	Diltiazem (L-type VDCCs), ω-conotoxin (N-type VDCCs)	Partial inhibition	[64]
		Mixture of diltiazem and ω-conotoxin (L-type and N-type VDCCs)	Full inhibition	[64]
	Rat hippocampal neurons	Nifedipine, Diltiazem (L-type VDCC)	Partial inhibition	[22,23,65]
	Rat hippocampal neurons	ω-conotoxin (N-type VDCCs)	Partial inhibition	[22,65]
	Rat hippocampal neurons	Mixture of diltiazem and ω-conotoxin	Full inhibition	[65]
		(L-type and N-type VDCCs)		
	Rat hippocampal neurons	ω-agatoxin (P/Q-type VDCCs)	Partial inhibition	[22]
	Rat hippocampal neurons	Mibefradil or pimozide (T-type VDCCs)	Full inhibition	[23]
	Rat hippocampal neurons	ω-conotoxin (N-type VDCCs)	No inhibition	[23]
	Rat hippocampal neurons	Loe908 or SKF-96365 (NSCCs)	Full inhibition	[23]
L1-CAM	PC12 cells	Verapamil, diltiazem, nifedipine or ω-conotoxin (L-type or N-type VDCCs)	Partial inhibition	[67]
	PC12 cells	Mixture of L-type and N-type VDCCs inhibitors	Full inhibition	[67]
	Rat dorsal root ganglion neurons	ω-conotoxin or verapamil (N-type or L-type VDCCs inhibitors)	Full inhibition	[31]
	Rat cerebellar neurons	Verapamil, diltiazem, nifedipine or ω-conotoxin (L-type or N-type VDCCs)	Partial inhibition	[67]
	Rat cerebellar neurons	Mixture of L-type and N-type VDCCs inhibitors	Full inhibition	[67]
	Mouse small cerebellar neurons	Verapamil, diltiazem, or nifedipine	Partial inhibition	[28]
		(L-type VDCCs)		
CHL1	Mouse hippocampal neurons	Nifedipine (L-type VDCCs)	Full inhibition	[33]
	Mouse hippocampal neurons	Pimozide (T-type VDCCs)	Full inhibition	[33]
Thy-1	PC12 cells	Diltiazem, nifedipine, verapamil or ω-conotoxin (L-type or N-type VDCCs)	Full inhibition	[68]
LAMP	Rat hippocampal neurons	Nifedipine (L-type VDCCs)	Partial inhibition	[36]
	Rat hippocampal neurons	ω-conotoxin (N-type VDCCs)	No inhibition	[36]
N-cadherin	PC12 cells	Diltiazem (L-type VDCCs), ω-conotoxin (N-type VDCCs)	Partial inhibition	[64]
	PC12 cells	Mixture of diltiazem and ω-conotoxin (L-type and N-type VDCCs)	Partial inhibition	[64]
	Chick ciliary ganglion neurons	Mixture of diltiazem and ω-conotoxin (L-type and N-type VDCCs)	No inhibition	[39]
β Integrin	Chick ciliary ganglion neurons	Mixture of diltiazem and ω-conotoxin (L-type and N-type VDCCs)	No inhibition	[39]

CAMs and cell type analyzed, inhibitors used and the impact of the inhibitors on the CAM-dependent neurite outgrowth are described.

Similarly to xxxxxxxxxxxIgSF CAMs, N-cadherin-mediated neurite outgrowth from PC12 cells has been shown to be inhibited by inhibitors of L- and N-type VDCCs in an additive manner [64]. However, inhibitors of L- and N-type VDCCs failed to block N-cadherin-dependent neurite outgrowth in ciliary ganglion neurons [39] indicating that other types of Ca^2+^ channels are involved in cadherin-dependent neurite outgrowth in these cells. Surprisingly, inhibitors of L- and N-type VDCCs diltiazem and ω-conotoxin had no effect on integrin-mediated laminin-induced neurite outgrowth in ciliary ganglion neurons [39]. Therefore, VDCCs required for integrin mediated neurite outgrowth remain to be identified.

### Potential mechanisms linking CAMs to Ca^2+^ channels

While data accumulated over the last two decades indicate that activation of CAMs induces an increase in intracellular Ca^2+^ levels in neurons, the mechanisms of this increase remain incompletely understood and probably involve a number of signaling cascades, which link CAMs to the sources of extra- and intracellular Ca^2+^ by changing the permeability of the respective channels.

**Figure 1:**
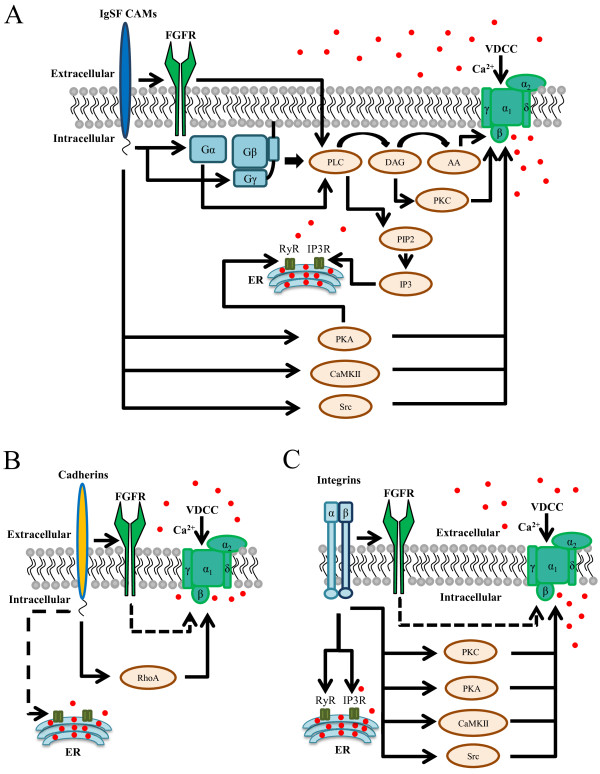
**Schematic representation of the possible mechanisms utilized by CAMs to induce an increase in the intracellular Ca**^**2+ **^**levels.** IgSF CAMs **(A)**, cadherins **(B)**, and integrins **(C)** can influence intracellular Ca^2+^ levels by inducing intracellular signaling cascades converging on VDCCs or inducing Ca^2+^ release from the internal stores (solid arrows). Known intermediate enzymes involved are also shown. Dashed arrows represent proposed pathways, which have not been experimentally analyzed yet. See text for details and references. FGFR - fibroblast growth factor receptor; VDCC - voltage dependent calcium channels with α_1_, α_2_, β, γ, and δ denoting the subunits of VDCC; G_α_, G_β_, and G_γ_ - subunits of the G-protein; PLC - phospholipase C; DAG - diacylycerol; AA - arachidonic acid; PKC - protein kinase C; Src - Src-family tyrosine kinase; PKA - protein kinase A; CaMKII - calcium/calmodulin-dependent kinase II; PIP2 - phosphatidylinositol 4, 5-bisphosphate; IP3 - inositol 1,4,5-triphosphate; IP3R - inositol triphosphate receptor; PKA - protein kinase A; RhoA – Ras homolog gene family A; RyR - ryanodine receptor; ER - endoplasmic reticulum.

A possibility that CAMs change permeability of VDCCs is supported by the studies on IgSF CAM L1 showing that Ca^2+^ influx via VDCCs in response to L1 activation occurs without changes in membrane voltage [37] indicating that L1 promotes Ca^2+^ influx via changing VDCCs properties. Probably the best characterized signaling pathway activated by IgSF CAMs to induce changes in intracellular Ca^2+^ levels involves the fibroblast growth factor receptor (FGFR) (Figure 1A). FGFR directly interacts with the members of the immunoglobulin superfamily NCAM [70,71], Nectin-1 [72], neuroplastin [35] and L1 [73,74]. It was proposed that activation of FGFR in this pathway is followed by activation of phospholipase C (PLC), which generates diacylglycerol (DAG), which is then converted into arachidonic acid (AA), which then activates VDCCs and subsequently induces Ca^2+^ influx [37,75]. In agreement with this model, ion influx through VDCCs in response to L1 activation was inhibited by a DAG lipase inhibitor and blocked in sensory neurons expressing dominant negative FGFR [37]. Further confirming this model, inhibitors of FGFR and PLC also reduced an increase in intracellular Ca^2+^ levels in response to NCAM activation in cultured hippocampal neurons [23].

Inhibitors of FGFR do not fully block NCAM-mediated increases in intracellular Ca^2+^ levels, suggesting that other factors also contribute to changes in the levels of intracellular Ca^2+^. Src-family tyrosine kinases were implicated since inhibitors of this family of tyrosine kinases partially reduced an increase in intracellular Ca^2+^ levels in response to activation of NCAM in neurons [23]. Src-family tyrosine kinases associate with and regulate the activity of L-type VDCCs [76], and are activated by different members of the immunoglobulin superfamily including NCAM [77] and L1 [78] (Figure 1A). Interestingly, NCAM and L1 act at different members of the Src-family tyrosine kinase family, fyn and src respectively [78-80]. It remains to be determined whether this influences the ability of NCAM and L1 to activate different VDCCs at the cell surface.

The G protein pathway is another pathway which may contribute to activation of VDCCs [81,82], possibly via inducing PLC activation [83] (Figure 1A). Pertussis toxin (PTX), an inhibitor of the G protein, inhibited Ca^2+^ influx in response to activation of NCAM in PC12 cells [24] and in response to NgCAM in avian forebrain neurons [32].

An increase in intracellular levels of Ca^2+^ in response to activation of IgSF CAMs at the cell surface has also been linked to activation of a number of Ca^2+^ dependent enzymes, such as protein kinase C (PKC) or calcium/calmodulin-dependent kinase II α (CaMKIIα), which are activated by NCAM [84-87]. It is therefore possible that PKC and CaMKII provide a positive feedback loop to increase the Ca^2+^ influx via VDCCs in response to activation of NCAM (Figure 1A). Interestingly, however, the long-term exposure of neurons to the PKC activator phorbol 12-myristate 13-acetate inhibited L1-depenent increases in intracellular Ca^2+^ levels [29,88], suggesting that PKC may also play a role in reducing intracellular Ca^2+^ levels following CAM activation.

There is also limited evidence on the interactions between IgSF CAMs and VDCCs in neurons. Both L-type and T-type VDCCs co-immunoprecipitated with NCAM from the mouse brain lysates indicating that NCAM forms a molecular complex with VDCCs [86]. In response to ligand binding, NCAM redistributes to lipid rafts [77,89], where VDCCs are also accumulated [86]. Whether the interactions between NCAM and VDCCs influence the permeability of VDCCs remains to be investigated.

IgSF CAM-activated signaling pathways can also play a central role in inducing Ca^2+^ release from internal stores. Inhibitors of PLC inhibited increases in the levels of intracellular Ca^2+^ in response to NCAM activation, suggesting that Ca^2+^ release from internal stores occurs in response to inositol-3-phosphate produced by PLC (Figure 1A). Additionally, activation of RyR via cAMP and PKA pathways may also result in Ca^2+^ release from internal stores [90] (Figure 1A). This CAM-dependent activation of RyR via cAMP and PKA pathways have been shown to have important consequences for neuronal behavior, such as turning of growth cones of growing neurites either towards the source of Ca^2+^ signal, which occurs on the L1- or N-cadherin substarte, or away from the source of Ca^2+^ signal which occurs on the laminin substrate in dorsal root ganglion neurons [90].

Similarly to IgSF members cadherins also interact with FGFR [91-93] (Figure 1B). This observation suggests that cadherins can activate signaling pathways which are similar to or partially overlap with signaling pathways activated by IgSF members to induce Ca^2+^ influx at the cell surface. N-cadherin dependent regulation of VDCCs involves, however, also a small GTPase RhoA [94,95] (Figure 1B).

FGFR also binds to integrins [96], and may be involved in integrin-dependent Ca^2+^ signalling (Figure 1C). Inhibition of Src family tyrosine kinases also partially reduced an increase in intracellular Ca^2+^ levels in response to activation integrins in neurons [47], suggesting that kinases of this family are involved (Figure 1C). Integrin-dependent activation of L-type VDCCs has also been shown to be dependent on PKA [47], which phosphorylates VDCCs and facilitates their function [97] (Figure 1C). Laminin-induced integrin-mediated increases in intracellular Ca^2+^ levels in growth cones were also blocked by inhibitors of PKC and CaMKIIα [43] (Figure 1C).

Integrins have also been found in a complex with VDCCs. Integrins containing α3 subunit are linked to VDCCs by laminin in the Torpedo electric organ synapses [98]. In cerebellar granular neurons, integrin α5β1 associates with short transient receptor potential channel 5 (TrpC5) [99]. TrpC channels are metabolically-activated Ca^2+^ channels, which are widely expressed in different tissues and cell types playing diverse physiological functions [100]. They also play a critical role in neuronal development (for extensive review see [101]) and neurite outgrowth in particular [99,102]. In non-neuronal human embryonic kidney HEK293 cells, α5β1-integrin has been shown to associate with L-type VDCCs [103], and ligand-dependent complex formation between β1-integrin and L-type VDCCs has been described in mouse embryonic stem cells [104]. Whether binding of integrins to VDCCs and TRPCs influences permeability of these channels remains however unknown.

## Conclusion

In conclusion, a number of studies indicate that CAMs play an important role in regulation of intracellular Ca^2+^ levels in neurons by acting at VDCCs and possibly other types of Ca^2+^ channels in the neuronal cell surface plasma membrane and in the intracellular Ca^2+^ stores. Not only VDCCs, but also other neuronal plasma membrane Ca^2+^ channels such as transient receptor potential channels, stretch-activated channels, and cyclic nucleotide-gated channels have been reported to play a role in neuronal development [105-107]. Direct links between CAMs and other types of Ca^2+^ channels in neurons remain however unknown. Current data indicate that CAMs activate Ca^2+^ channels by inducing intracellular signaling cascades which can either activate or remove inhibition of Ca^2+^ channels to induce an increase in intracellular Ca^2+^ levels. It remains to be investigated whether formation of the molecular complexes between CAMs and Ca^2+^ channels directly influences the activity of the latter.

Most of the previous work was performed using artificial stimulation protocols by applying soluble ligands in the culture medium and monitoring bulk changes in intracellular Ca^2+^ levels. Better imaging technologies which appeared recently may help to investigate the dynamics of local CAM-dependent Ca^2+^ changes occurring during cell-to-cell contact formation, and particularly during synapse formation. A combination of such technologies with biochemical analysis and optical imaging of the synapse enriched cytoskeleton components and enzymes may provide valuable information about the mechanisms of the molecular rearrangements accompanying contact maturation. The development of genetically encoded Ca^2+^ reporters [108-111] with a better defined subcellular localization as compared to chemical dyes used in previous studies may also allow monitoring intracellular Ca^2+^ levels in CAM-enriched membrane microdomains in different neuronal compartments, which is a promising new direction for further research.

## Abbreviations

AA: Arachidonic acid; Aβ: Amyloid beta; AM: Acetoxymethyl ester; APP: Amyloid precursor protein; CAMs: Cell adhesion molecules; CaMKIIα: Calcium/calmodulin-dependent kinase II α; CHL1: Close homolog of L1; CHO: Chinese hamster ovary cells; CNS: Central nervous system; DAG: Diacylglycerol; ER: Endoplasmic reticulum; FGFR: Fibroblast growth factor receptor; HEK 293 cells: Human embryonic kidney 293 cells; IP3: Inositol 1,4,5-triphosphate; IP3R: Inositol 1,4,5-triphosphate gated receptor; LAMP: Limbic system-associated membrane protein; NCAM: Neural cell adhesion molecule; NgCAM: Neuron-glia cell adhesion molecule; NMDA: N-methyl-D-aspartate; Np: Neuroplastin; NSCCs: Nonselective cation channels; PLC: Phospholipase C; PKA: Protein kinase A; PKC: Protein kinase C; PTX: Pertussis toxin; PrP: Prion protein; RGD: Arginine-Glycine-Aspartic acid; RyR: Ryanodine receptor; TrpC5: Transient receptor potential channel 5; VDCCs: Voltage-dependent calcium channels.

## Competing interests

The authors declare that they have no competing interests.

## Authors’ contributions

LS, IL and VS have performed literature search and analysis and written the manuscript. All authors read and approved the final manuscript.

## References

[B1] Tessier-Lavigne M Goodman CS The molecular biology of axon guidance Science 1996 11 1123 1133 10.1126/science.274.5290.1123 8895455

[B2] Walsh FS Meiri K Doherty P Cell signalling and CAM-mediated neurite outgrowth Soc Gen Physiol Ser 1997 11 221 226 9210232

[B3] Blackmore M Letourneau PC L1, beta1 integrin, and cadherins mediate axonal regeneration in the embryonic spinal cord J Neurobiol 2006 11 1564 1583 10.1002/neu.20311 17058193

[B4] Nacher J Guirado R Castillo-Gomez E Structural plasticity of interneurons in the adult brain: role of PSA-NCAM and implications for psychiatric disorders Neurochem Res 2013 11 1122 1133 10.1007/s11064-013-0977-4 23354722

[B5] Hansen SM Berezin V Bock E Signaling mechanisms of neurite outgrowth induced by the cell adhesion molecules NCAM and N-cadherin Cell Mol Life Sci 2008 11 3809 3821 10.1007/s00018-008-8290-0 18791849 PMC11131707

[B6] Rutishauser U Polysialic acid in the plasticity of the developing and adult vertebrate nervous system Nat Rev Neurosci 2008 11 26 35 10.1038/nrn2285 18059411

[B7] Cavallaro U Dejana E Adhesion molecule signalling: not always a sticky business Nat Rev Mol Cell Biol 2011 11 189 197 21346732 10.1038/nrm3068

[B8] Gahmberg CG Fagerholm SC Nurmi SM Chavakis T Marchesan S Gronholm M Regulation of integrin activity and signalling Biochim Biophys Acta 2009 11 431 444 10.1016/j.bbagen.2009.03.007 19289150 PMC2734279

[B9] Aricescu AR Jones EY Immunoglobulin superfamily cell adhesion molecules: zippers and signals Curr Opin Cell Biol 2007 11 543 550 10.1016/j.ceb.2007.09.010 17935964

[B10] Dalva MB McClelland AC Kayser MS Cell adhesion molecules: signalling functions at the synapse Nat Rev Neurosci 2007 11 206 220 17299456 10.1038/nrn2075 PMC4756920

[B11] Doherty P Williams G Williams EJ CAMs and axonal growth: a critical evaluation of the role of calcium and the MAPK cascade Mol Cell Neurosci 2000 11 283 295 10.1006/mcne.2000.0907 11085868

[B12] Bedlack RS Jr Wei M Loew LM Localized membrane depolarizations and localized calcium influx during electric field-guided neurite growth Neuron 1992 11 393 403 10.1016/0896-6273(92)90178-G 1524823

[B13] Rehder V Kater SB Regulation of neuronal growth cone filopodia by intracellular calcium J Neurosci 1992 11 3175 3186 1494951 10.1523/JNEUROSCI.12-08-03175.1992 PMC6575658

[B14] Henley JR Huang KH Wang D Poo MM Calcium mediates bidirectional growth cone turning induced by myelin-associated glycoprotein Neuron 2004 11 909 916 10.1016/j.neuron.2004.11.030 15603734 PMC3121244

[B15] Zheng JQ Poo MM Calcium signaling in neuronal motility Annu Rev Cell Dev Biol 2007 11 375 404 10.1146/annurev.cellbio.23.090506.123221 17944572

[B16] Henley J Poo MM Guiding neuronal growth cones using Ca2+ signals Trends Cell Biol 2004 11 320 330 10.1016/j.tcb.2004.04.006 15183189 PMC3115711

[B17] Gomez TM Zheng JQ The molecular basis for calcium-dependent axon pathfinding Nat Rev Neurosci 2006 11 115 125 10.1038/nrn1844 16429121

[B18] Maness PF Schachner M Neural recognition molecules of the immunoglobulin superfamily: signaling transducers of axon guidance and neuronal migration Nat Neurosci 2007 11 19 26 10.1038/nn1827 17189949

[B19] Ditlevsen DK Povlsen GK Berezin V Bock E NCAM-induced intracellular signaling revisited J Neurosci Res 2008 11 727 743 10.1002/jnr.21551 17975827

[B20] Kraev I Henneberger C Rossetti C Conboy L Kohler LB Fantin M Jennings A Venero C Popov V Rusakov D A peptide mimetic targeting trans-homophilic NCAM binding sites promotes spatial learning and neural plasticity in the hippocampus PloS One 2011 11 e23433 10.1371/journal.pone.0023433 21887252 PMC3160849

[B21] Von Bohlen Und Halbach F Taylor J Schachner M Cell Type-specific Effects of the Neural Adhesion Molecules L1 and N-CAM on Diverse Second Messenger Systems Eur J Neurosci 1992 11 896 909 10.1111/j.1460-9568.1992.tb00116.x 12106425

[B22] Ronn LC Dissing S Holm A Berezin V Bock E Increased intracellular calcium is required for neurite outgrowth induced by a synthetic peptide ligand of NCAM FEBS Lett 2002 11 60 66 10.1016/S0014-5793(02)02644-3 11997018

[B23] Kiryushko D Korshunova I Berezin V Bock E Neural cell adhesion molecule induces intracellular signaling via multiple mechanisms of Ca2+ homeostasis Mol Biol Cell 2006 11 2278 2286 10.1091/mbc.E05-10-0987 16510522 PMC1446100

[B24] Schuch U Lohse MJ Schachner M Neural cell adhesion molecules influence second messenger systems Neuron 1989 11 13 20 10.1016/0896-6273(89)90111-6 2559759

[B25] Frei T von Bohlen und Halbach F Wille W Schachner M Different extracellular domains of the neural cell adhesion molecule (N-CAM) are involved in different functions J Cell Biol 1992 11 177 194 10.1083/jcb.118.1.177 1618903 PMC2289517

[B26] Brummendorf T Rathjen FG Structure/function relationships of axon-associated adhesion receptors of the immunoglobulin superfamily Curr Opin Neurobiol 1996 11 584 593 10.1016/S0959-4388(96)80089-4 8937821

[B27] Grumet M Cell adhesion molecules and their subgroups in the nervous system Curr Opin Neurobiol 1991 11 370 376 10.1016/0959-4388(91)90055-C 1821679

[B28] Appel F Holm J Conscience JF von Bohlen und Halbach F Faissner A James P Schachner M Identification of the border between fibronectin type III homologous repeats 2 and 3 of the neural cell adhesion molecule L1 as a neurite outgrowth promoting and signal transducing domain J Neurobiol 1995 11 297 312 10.1002/neu.480280304 8568512

[B29] Itoh K Kawamura H Asou H A novel monoclonal antibody against carbohydrates of L1 cell adhesion molecule causes an influx of calcium in cultured cortical neurons Brain Res 1992 11 233 240 10.1016/0006-8993(92)90949-A 1504802

[B30] Asou H Monoclonal antibody that recognizes the carbohydrate portion of cell adhesion molecule L1 influences calcium current in cultured neurons J Cell Physiol 1992 11 313 320 10.1002/jcp.1041530211 1385450

[B31] Harper SJ Bolsover SR Walsh FS Doherty P Neurite outgrowth stimulated by L1 requires calcium influx into neurons but is not associated with changes in steady state levels of calcium in growth cones Cell Adhes Commun 1994 11 441 453 10.3109/15419069409004454 7842257

[B32] Goldman SA Williams S Barami K Lemmon V Nedergaard M Transient coupling of Ng-CAM expression to NgCAM-dependent calcium signaling during migration of new neurons in the adult songbird brain Mol Cell Neurosci 1996 11 29 45 8812057 10.1006/mcne.1996.0003

[B33] Tian N Leshchyns’ka I Welch JH Diakowski W Yang H Schachner M Sytnyk V Lipid raft-dependent endocytosis of close homolog of adhesion molecule L1 (CHL1) promotes neuritogenesis J Biol Chem 2012 11 44447 44463 10.1074/jbc.M112.394973 23144456 PMC3531758

[B34] Owczarek S Soroka V Kiryushko D Larsen MH Yuan Q Sandi C Berezin V Bock E Neuroplastin-65 and a mimetic peptide derived from its homophilic binding site modulate neuritogenesis and neuronal plasticity J Neurochem 2011 11 984 994 10.1111/j.1471-4159.2011.07269.x 21480899

[B35] Owczarek S Kiryushko D Larsen MH Kastrup JS Gajhede M Sandi C Berezin V Bock E Soroka V Neuroplastin-55 binds to and signals through the fibroblast growth factor receptor FASEB J 2010 11 1139 1150 10.1096/fj.09-140509 19952283

[B36] Zhukareva V Chernevskaya N Pimenta A Nowycky M Levitt P Limbic system-associated membrane protein (LAMP) induces neurite outgrowth and intracellular Ca2+ increase in primary fetal neurons Mol Cell Neurosci 1997 11 43 55 10.1006/mcne.1997.0639 9361287

[B37] Archer FR Doherty P Collins D Bolsover SR CAMs and FGF cause a local submembrane calcium signal promoting axon outgrowth without a rise in bulk calcium concentration Eur J Neurosci 1999 11 3565 3573 10.1046/j.1460-9568.1999.00773.x 10564364

[B38] Shima Y Kawaguchi SY Kosaka K Nakayama M Hoshino M Nabeshima Y Hirano T Uemura T Opposing roles in neurite growth control by two seven-pass transmembrane cadherins Nat Neurosci 2007 11 963 969 10.1038/nn1933 17618280

[B39] Bixby JL Grunwald GB Bookman RJ Ca2+ influx and neurite growth in response to purified N-cadherin and laminin J Cell Biol 1994 11 1461 1475 10.1083/jcb.127.5.1461 7962102 PMC2120265

[B40] Chadborn N Eickholt B Doherty P Bolsover S Direct measurement of local raised subplasmalemmal calcium concentrations in growth cones advancing on an N-cadherin substrate Eur J Neurosci 2002 11 1891 1898 10.1046/j.1460-9568.2002.02033.x 12099895

[B41] Marrs GS Theisen CS Bruses JL N-cadherin modulates voltage activated calcium influx via RhoA, p120-catenin, and myosin-actin interaction Mol Cell Neurosci 2009 11 390 400 10.1016/j.mcn.2008.12.007 19162191 PMC2883866

[B42] Shima Y Copeland NG Gilbert DJ Jenkins NA Chisaka O Takeichi M Uemura T Differential expression of the seven-pass transmembrane cadherin genes Celsr1-3 and distribution of the Celsr2 protein during mouse development Dev Dyn 2002 11 321 332 10.1002/dvdy.10054 11891983

[B43] Kuhn TB Williams CV Dou P Kater SB Laminin directs growth cone navigation via two temporally and functionally distinct calcium signals J Neurosci 1998 11 184 194 9412499 10.1523/JNEUROSCI.18-01-00184.1998 PMC6793400

[B44] Gomez TM Robles E Poo M Spitzer NC Filopodial calcium transients promote substrate-dependent growth cone turning Science 2001 11 1983 1987 10.1126/science.1056490 11239161

[B45] Lin CY Hilgenberg LG Smith MA Lynch G Gall CM Integrin regulation of cytoplasmic calcium in excitatory neurons depends upon glutamate receptors and release from intracellular stores Mol Cell Neurosci 2008 11 770 780 10.1016/j.mcn.2008.01.001 18289871 PMC2396149

[B46] Watson PM Humphries MJ Relton J Rothwell NJ Verkhratsky A Gibson RM Integrin-binding RGD peptides induce rapid intracellular calcium increases and MAPK signaling in cortical neurons Mol Cell Neurosci 2007 11 147 154 10.1016/j.mcn.2006.10.007 17150373

[B47] Gui P Wu X Ling S Stotz SC Winkfein RJ Wilson E Davis GE Braun AP Zamponi GW Davis MJ Integrin receptor activation triggers converging regulation of Cav1.2 calcium channels by c-Src and protein kinase A pathways J Biol Chem 2006 11 14015 14025 10.1074/jbc.M600433200 16554304

[B48] Wildering WC Hermann PM Bulloch AG Rapid neuromodulatory actions of integrin ligands J Neurosci 2002 11 2419 2426 11923405 10.1523/JNEUROSCI.22-07-02419.2002 PMC6758314

[B49] Cingolani LA Thalhammer A Yu LM Catalano M Ramos T Colicos MA Goda Y Activity-dependent regulation of synaptic AMPA receptor composition and abundance by beta3 integrins Neuron 2008 11 749 762 10.1016/j.neuron.2008.04.011 18549786 PMC2446609

[B50] Shaked GM Chauv S Ubhi K Hansen LA Masliah E Interactions between the amyloid precursor protein C-terminal domain and G proteins mediate calcium dysregulation and amyloid beta toxicity in Alzheimer’s disease FEBS J 2009 11 2736 2751 10.1111/j.1742-4658.2009.06997.x 19368557 PMC2838422

[B51] Linde CI Baryshnikov SG Mazzocco-Spezzia A Golovina VA Dysregulation of Ca2+ signaling in astrocytes from mice lacking amyloid precursor protein Am J Physiol Cell Physiol 2011 11 C1502 C1512 10.1152/ajpcell.00379.2010 21368296 PMC3118622

[B52] Whatley SA Powell JF Politopoulou G Campbell IC Brammer MJ Percy NS Regulation of intracellular free calcium levels by the cellular prion protein Neuroreport 1995 11 2333 2337 10.1097/00001756-199511270-00015 8747148

[B53] Santos TG Beraldo FH Hajj GN Lopes MH Roffe M Lupinacci FC Ostapchenko VG Prado VF Prado MA Martins VR Laminin-gamma1 chain and stress inducible protein 1 synergistically mediate PrPC-dependent axonal growth via Ca2+ mobilization in dorsal root ganglia neurons J Neurochem 2013 11 210 223 10.1111/jnc.12091 23145988

[B54] Herms JW Korte S Gall S Schneider I Dunker S Kretzschmar HA Altered intracellular calcium homeostasis in cerebellar granule cells of prion protein-deficient mice J Neurochem 2000 11 1487 1492 10987828 10.1046/j.1471-4159.2000.0751487.x

[B55] Fuhrmann M Bittner T Mitteregger G Haider N Moosmang S Kretzschmar H Herms J Loss of the cellular prion protein affects the Ca2+ homeostasis in hippocampal CA1 neurons J Neurochem 2006 11 1876 1885 10.1111/j.1471-4159.2006.04011.x 16945105

[B56] Missler M Zhang W Rohlmann A Kattenstroth G Hammer RE Gottmann K Sudhof TC Alpha-neurexins couple Ca2+ channels to synaptic vesicle exocytosis Nature 2003 11 939 948 10.1038/nature01755 12827191

[B57] Dolphin AC Calcium channel diversity: multiple roles of calcium channel subunits Curr Opin Neurobiol 2009 11 237 244 10.1016/j.conb.2009.06.006 19559597

[B58] Rajakulendran S Kaski D Hanna MG Neuronal P/Q-type calcium channel dysfunction in inherited disorders of the CNS Nat Rev Neurol 2012 11 86 96 10.1038/nrneurol.2011.228 22249839

[B59] Calin-Jageman I Lee A Ca(v)1 L-type Ca2+ channel signaling complexes in neurons J Neurochem 2008 11 573 583 10.1111/j.1471-4159.2008.05286.x 18266933

[B60] Evans RM Zamponi GW Presynaptic Ca2+ channels–integration centers for neuronal signaling pathways Trends Neurosci 2006 11 617 624 10.1016/j.tins.2006.08.006 16942804

[B61] Neher E Sakaba T Multiple roles of calcium ions in the regulation of neurotransmitter release Neuron 2008 11 861 872 10.1016/j.neuron.2008.08.019 18817727

[B62] Cataldi M The changing landscape of voltage-gated calcium channels in neurovascular disorders and in neurodegenerative diseases Curr Neuropharmacol 2013 11 276 297 10.2174/1570159X11311030004 24179464 PMC3648780

[B63] Lipscombe D Helton TD Xu W L-type calcium channels: the low down J Neurophysiol 2004 11 2633 2641 10.1152/jn.00486.2004 15486420

[B64] Doherty P Ashton SV Moore SE Walsh FS Morphoregulatory activities of NCAM and N-cadherin can be accounted for by G protein-dependent activation of L- and N-type neuronal Ca2+ channels Cell 1991 11 21 33 10.1016/0092-8674(91)90569-K 1680564

[B65] Doherty P Skaper SD Moore SE Leon A Walsh FS A developmentally regulated switch in neuronal responsiveness to NCAM and N-cadherin in the rat hippocampus Development 1992 11 885 892 1425359 10.1242/dev.115.3.885

[B66] Chernyshova Y Leshchyns’ka I Hsu SC Schachner M Sytnyk V The neural cell adhesion molecule promotes FGFR-dependent phosphorylation and membrane targeting of the exocyst complex to induce exocytosis in growth cones J Neurosci 2011 11 3522 3535 10.1523/JNEUROSCI.3109-10.2011 21389209 PMC6622784

[B67] Williams EJ Doherty P Turner G Reid RA Hemperly JJ Walsh FS Calcium influx into neurons can solely account for cell contact-dependent neurite outgrowth stimulated by transfected L1 J Cell Biol 1992 11 883 892 10.1083/jcb.119.4.883 1429842 PMC2289701

[B68] Doherty P Singh A Rimon G Bolsover SR Walsh FS Thy-1 antibody-triggered neurite outgrowth requires an influx of calcium into neurons via N- and L-type calcium channels J Cell Biol 1993 11 181 189 10.1083/jcb.122.1.181 8100230 PMC2119600

[B69] Ohbayashi K Fukura H Inoue HK Komiya Y Igarashi M Stimulation of L-type Ca2+ channel in growth cones activates two independent signaling pathways J Neurosci Res 1998 11 682 696 10.1002/(SICI)1097-4547(19980315)51:6<682::AID-JNR3>3.0.CO;2-7 9545083

[B70] Kiselyov VV Skladchikova G Hinsby AM Jensen PH Kulahin N Soroka V Pedersen N Tsetlin V Poulsen FM Berezin V Bock E Structural basis for a direct interaction between FGFR1 and NCAM and evidence for a regulatory role of ATP Structure 2003 11 691 701 10.1016/S0969-2126(03)00096-0 12791257

[B71] Kochoyan A Poulsen FM Berezin V Bock E Kiselyov VV Structural basis for the activation of FGFR by NCAM Protein Sci 2008 11 1698 1705 10.1110/ps.035964.108 18593816 PMC2548372

[B72] Bojesen KB Clausen O Rohde K Christensen C Zhang L Li S Kohler L Nielbo S Nielsen J Gjorlund MD Nectin-1 binds and signals through the fibroblast growth factor receptor J Biol Chem 2012 11 37420 37433 10.1074/jbc.M112.345215 22955284 PMC3481338

[B73] Williams EJ Furness J Walsh FS Doherty P Activation of the FGF receptor underlies neurite outgrowth stimulated by L1, N-CAM, and N-cadherin Neuron 1994 11 583 594 10.1016/0896-6273(94)90027-2 7917292

[B74] Kulahin N Li S Hinsby A Kiselyov V Berezin V Bock E Fibronectin type III (FN3) modules of the neuronal cell adhesion molecule L1 interact directly with the fibroblast growth factor (FGF) receptor Mol Cell Neurosci 2008 11 528 536 10.1016/j.mcn.2007.12.001 18222703

[B75] Williams EJ Walsh FS Doherty P The production of arachidonic acid can account for calcium channel activation in the second messenger pathway underlying neurite outgrowth stimulated by NCAM, N-cadherin, and L1 J Neurochem 1994 11 1231 1234 8113807 10.1046/j.1471-4159.1994.62031231.x

[B76] Hu XQ Singh N Mukhopadhyay D Akbarali HI Modulation of voltage-dependent Ca2+ channels in rabbit colonic smooth muscle cells by c-Src and focal adhesion kinase J Biol Chem 1998 11 5337 5342 10.1074/jbc.273.9.5337 9478993

[B77] Bodrikov V Leshchyns’ka I Sytnyk V Overvoorde J den Hertog J Schachner M RPTPalpha is essential for NCAM-mediated p59fyn activation and neurite elongation J Cell Biol 2005 11 127 139 15623578 10.1083/jcb.200405073 PMC2171675

[B78] Schmid RS Pruitt WM Maness PF A MAP kinase-signaling pathway mediates neurite outgrowth on L1 and requires Src-dependent endocytosis J Neurosci 2000 11 4177 4188 10818153 10.1523/JNEUROSCI.20-11-04177.2000 PMC6772629

[B79] Beggs HE Soriano P Maness PF NCAM-dependent neurite outgrowth is inhibited in neurons from Fyn-minus mice J Cell Biol 1994 11 825 833 7962063 10.1083/jcb.127.3.825 PMC2120232

[B80] Beggs HE Baragona SC Hemperly JJ Maness PF NCAM140 interacts with the focal adhesion kinase p125(fak) and the SRC-related tyrosine kinase p59(fyn) J Biol Chem 1997 11 8310 8319 10.1074/jbc.272.13.8310 9079653

[B81] Tedford HW Zamponi GW Direct G protein modulation of Cav2 calcium channels Pharmacol Rev 2006 11 837 862 10.1124/pr.58.4.11 17132857

[B82] Strock J Diverse-Pierluissi MA Ca2+ channels as integrators of G protein-mediated signaling in neurons Mol Pharmacol 2004 11 1071 1076 10.1124/mol.104.002261 15269290

[B83] Bunney TD Katan M PLC regulation: emerging pictures for molecular mechanisms Trends Biochem Sci 2011 11 88 96 10.1016/j.tibs.2010.08.003 20870410

[B84] Kolkova K Stensman H Berezin V Bock E Larsson C Distinct roles of PKC isoforms in NCAM-mediated neurite outgrowth J Neurochem 2005 11 886 894 10.1111/j.1471-4159.2004.02919.x 15686491

[B85] Leshchyns’ka I Sytnyk V Morrow JS Schachner M Neural cell adhesion molecule (NCAM) association with PKCbeta2 via betaI spectrin is implicated in NCAM-mediated neurite outgrowth J Cell Biol 2003 11 625 639 10.1083/jcb.200303020 12743109 PMC2172933

[B86] Bodrikov V Sytnyk V Leshchyns’ka I den Hertog J Schachner M NCAM induces CaMKIIalpha-mediated RPTPalpha phosphorylation to enhance its catalytic activity and neurite outgrowth J Cell Biol 2008 11 1185 1200 10.1083/jcb.200803045 18809727 PMC2542478

[B87] Sytnyk V Leshchyns’ka I Nikonenko AG Schachner M NCAM promotes assembly and activity-dependent remodeling of the postsynaptic signaling complex J Cell Biol 2006 11 1071 1085 10.1083/jcb.200604145 17000882 PMC2064397

[B88] Kadmon G von Bohlen und Halbach F Horstkorte R Eckert M Altevogt P Schachner M Evidence for cis interaction and cooperative signalling by the heat-stable antigen nectadrin (murine CD24) and the cell adhesion molecule L1 in neurons Eur J Neurosci 1995 11 993 1004 10.1111/j.1460-9568.1995.tb01087.x 7613634

[B89] Santuccione A Sytnyk V Leshchyns’ka I Schachner M Prion protein recruits its neuronal receptor NCAM to lipid rafts to activate p59fyn and to enhance neurite outgrowth J Cell Biol 2005 11 341 354 10.1083/jcb.200409127 15851519 PMC2171870

[B90] Ooashi N Futatsugi A Yoshihara F Mikoshiba K Kamiguchi H Cell adhesion molecules regulate Ca2 + -mediated steering of growth cones via cyclic AMP and ryanodine receptor type 3 J Cell Biol 2005 11 1159 1167 10.1083/jcb.200503157 16172206 PMC2171540

[B91] Utton MA Eickholt B Howell FV Wallis J Doherty P Soluble N-cadherin stimulates fibroblast growth factor receptor dependent neurite outgrowth and N-cadherin and the fibroblast growth factor receptor co-cluster in cells J Neurochem 2001 11 1421 1430 10.1046/j.1471-4159.2001.00140.x 11238727

[B92] Sanchez-Heras E Howell FV Williams G Doherty P The fibroblast growth factor receptor acid box is essential for interactions with N-cadherin and all of the major isoforms of neural cell adhesion molecule J Biol Chem 2006 11 35208 35216 10.1074/jbc.M608655200 17005551

[B93] Suyama K Shapiro I Guttman M Hazan RB A signaling pathway leading to metastasis is controlled by N-cadherin and the FGF receptor Cancer Cell 2002 11 301 314 10.1016/S1535-6108(02)00150-2 12398894

[B94] Piccoli G Rutishauser U Bruses JL N-cadherin juxtamembrane domain modulates voltage-gated Ca2+ current via RhoA GTPase and Rho-associated kinase J Neurosci 2004 11 10918 10923 10.1523/JNEUROSCI.4020-04.2004 15574742 PMC6730207

[B95] White MG Crumling MA Meriney SD Developmental changes in calcium current pharmacology and somatostatin inhibition in chick parasympathetic neurons J Neurosci 1997 11 6302 6313 9236240 10.1523/JNEUROSCI.17-16-06302.1997 PMC6568329

[B96] Toledo MS Suzuki E Handa K Hakomori S Effect of ganglioside and tetraspanins in microdomains on interaction of integrins with fibroblast growth factor receptor J Biol Chem 2005 11 16227 16234 10.1074/jbc.M413713200 15710618

[B97] Carbone E Carabelli V Cesetti T Baldelli P Hernandez-Guijo JM Giusta L G-protein- and cAMP-dependent L-channel gating modulation: a manyfold system to control calcium entry in neurosecretory cells Pflugers Arch 2001 11 801 813 10.1007/s004240100607 11680611

[B98] Carlson SS Valdez G Sanes JR Presynaptic calcium channels and alpha3-integrins are complexed with synaptic cleft laminins, cytoskeletal elements and active zone components J Neurochem 2010 11 654 666 10.1111/j.1471-4159.2010.06965.x 20731762 PMC2970707

[B99] Wu G Lu ZH Obukhov AG Nowycky MC Ledeen RW Induction of calcium influx through TRPC5 channels by cross-linking of GM1 ganglioside associated with alpha5beta1 integrin initiates neurite outgrowth J Neurosci 2007 11 7447 7458 10.1523/JNEUROSCI.4266-06.2007 17626205 PMC6672619

[B100] Gees M Colsoul B Nilius B The role of transient receptor potential cation channels in Ca2+ signaling Cold Spring Harb Perspect Biol 2010 11 a003962 20861159 10.1101/cshperspect.a003962 PMC2944357

[B101] Bollimuntha S Selvaraj S Singh BB Emerging roles of canonical TRP channels in neuronal function Adv Exp Med Biol 2011 11 573 593 10.1007/978-94-007-0265-3_31 21290317 PMC3045772

[B102] Ramsey IS Delling M Clapham DE An introduction to TRP channels Annu Rev Physiol 2006 11 619 647 10.1146/annurev.physiol.68.040204.100431 16460286

[B103] Chao JT Gui P Zamponi GW Davis GE Davis MJ Spatial association of the Cav1.2 calcium channel with alpha5beta1-integrin Am J Physiol Cell Physiol 2011 11 C477 C489 10.1152/ajpcell.00171.2010 21178109 PMC3063962

[B104] Suh HN Han HJ Fibronectin-induced VEGF receptor and calcium channel transactivation stimulate GLUT-1 synthesis and trafficking through PPARgamma and TC10 in mouse embryonic stem cells Stem Cell Res 2013 11 371 386 10.1016/j.scr.2013.01.008 23455393

[B105] Jacques-Fricke BT Seow Y Gottlieb PA Sachs F Gomez TM Ca2+ influx through mechanosensitive channels inhibits neurite outgrowth in opposition to other influx pathways and release from intracellular stores J Neurosci 2006 11 5656 5664 10.1523/JNEUROSCI.0675-06.2006 16723522 PMC6675278

[B106] Kerstein PC Jacques-Fricke BT Rengifo J Mogen BJ Williams JC Gottlieb PA Sachs F Gomez TM Mechanosensitive TRPC1 channels promote calpain proteolysis of talin to regulate spinal axon outgrowth J Neurosci 2013 11 273 285 10.1523/JNEUROSCI.2142-12.2013 23283340 PMC3539200

[B107] Togashi K von Schimmelmann MJ Nishiyama M Lim CS Yoshida N Yun B Molday RS Goshima Y Hong K Cyclic GMP-gated CNG channels function in Sema3A-induced growth cone repulsion Neuron 2008 11 694 707 10.1016/j.neuron.2008.03.017 18549782

[B108] Zhao Y Araki S Wu J Teramoto T Chang YF Nakano M Abdelfattah AS Fujiwara M Ishihara T Nagai T Campbell RE An expanded palette of genetically encoded Ca(2)(+) indicators Science 2011 11 1888 1891 10.1126/science.1208592 21903779 PMC3560286

[B109] Shigetomi E Kracun S Khakh BS Monitoring astrocyte calcium microdomains with improved membrane targeted GCaMP reporters Neuron Glia Biol 2010 11 183 191 10.1017/S1740925X10000219 21205365 PMC3136572

[B110] Chen TW Wardill TJ Sun Y Pulver SR Renninger SL Baohan A Schreiter ER Kerr RA Orger MB Jayaraman V Ultrasensitive fluorescent proteins for imaging neuronal activity Nature 2013 11 295 300 10.1038/nature12354 23868258 PMC3777791

[B111] Akerboom J Chen TW Wardill TJ Tian L Marvin JS Mutlu S Calderon NC Esposti F Borghuis BG Sun XR Optimization of a GCaMP calcium indicator for neural activity imaging J Neurosci 2012 11 13819 13840 10.1523/JNEUROSCI.2601-12.2012 23035093 PMC3482105

